# Hydrovoltaic Power Generation Depend on Wettability at the Liquid–Solid Interface: Mechanisms, Materials, and Applications With Various Resource

**DOI:** 10.1002/EXP.70007

**Published:** 2025-02-04

**Authors:** Yejin Lee, Minwoo Lee, Junwoo Lee, Ho Won Jang, Ji‐Soo Jang

**Affiliations:** ^1^ Electronic and Hybrid Materials Research Center Korea Institute of Science and Technology (KIST) Seoul Republic of Korea; ^2^ Department of Materials Science and Engineering, Research Institute of Advanced Materials Seoul National University Seoul Republic of Korea; ^3^ Department of Materials Science and Engineering Korea University Seoul Republic of Korea; ^4^ Department of Molecular Science and Technology Ajou University Suwon Republic of Korea; ^5^ Advanced Institute of Convergence Technology Seoul National University Suwon Republic of Korea

**Keywords:** energy harvesting, water energy harvesting, hydrovoltaic power generation

## Abstract

With the global increase in energy consumption, there is a growing demand for green energy production, which has prompted the development of novel renewable energy sources. Recently, significant momentum has been observed in research on new energy harvesting methods suitable for small devices. In this context, hydrovoltaic power generation, utilizing water due to its ubiquitous presence and easy availability, has emerged as a promising technology. Hydrovoltaic power generation operates by converting the potential energy of water into electrical energy through the interaction between water and materials capable of inducing an electrical potential gradient. The control of material surface wettability, which determines the interaction with water, plays a crucial role in enhancing the electrical output and long‐term stability of power generation systems. This review categorizes the mechanisms of hydrovoltaic power generation into flow and diffusion mechanisms, discussing respective case studies based on hydrophobic and hydrophilic substrates. Additionally, representative materials used in hydrovoltaic power generation are discussed and the potential to expand this technology across various fields based on the diverse resources of water is demonstrated. The review concludes with future perspectives, highlighting the applications of hydrovoltaic power generation across multiple domains and outlining directions for future research and development.

## Graphical abstract

This study explores hydrovoltaic power generation, a promising energy harvesting technology that utilizes water as a renewable energy source to address climate change. Focusing on the interfacial characteristics between solid surfaces and water, the research highlights the underlying mechanisms, material designs, and potential applications of this technology. The findings aim to advance sustainable energy solutions through innovative approaches to hydrovoltaic power generation.

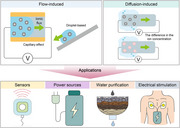

## 1 Introduction

Hydrovoltaic power generation is an innovative method of generating sustainable electricity by harnessing the natural processes involving water, such as evaporation and flow, offering the promise of clean and renewable power [1]. This method addresses the global increase in energy consumption and the corresponding need for increased energy production. While various renewable energy sources such as traditional hydro [2], wind [3], tidal [4], and solar power [5] are being utilized, challenges persist in securing stable energy supply, achieving high energy efficiency relative to installation costs, and minimizing environmental pollution during land acquisition [6]. Recently, there has been significant research into small‐scale power generation devices that can operate the Internet of Things (IoT) and wearable devices [7]. However, power generation devices such as solar [8], thermoelectric [9], piezoelectric [10], and triboelectric generators [11] are limited in terms of cost‐effectiveness and spatial constraints due to their operation being restricted to specific environmental conditions. Therefore, it is essential to explore the unique power generation methods of hydrovoltaic power generation to address the limitations of traditional power generation methods.

Hydrovoltaic power generation technology leverages the interaction between water and specific nanomaterials to convert kinetic or potential energy into electrical energy [12]. For flow‐induced electricity, water flowing over nanomaterial surfaces can generate electricity [13]. As water molecules interact with the material, electron flow is induced, generating a current. In the case of the diffusion‐induced method, when water evaporates from a surface coated with a nanomaterial, electrons are caused to move, generating a current [14]. This phenomenon occurs because the evaporating water molecules carry away a portion of the charge, creating a potential difference. Based on these principles of power generation, there is a need to classify hydrovoltaic power generation in terms of materials and water sources to facilitate the research and application of next‐generation power devices aimed at achieving high efficiency and environmental friendliness.

First, power generation properties can vary depending on the hydrophilicity or hydrophobicity of the substrate. A hydrophilic substrate interacts strongly with water, allowing water molecules to adhere well to the surface. Consequently, water spreads widely over the hydrophilic surface, resulting in a uniform distribution of water molecules across the entire substrate [15]. The use of hydrophilic substrates results in consistent and stable electricity generation due to the uniform distribution of water and higher evaporation rates, which can enhance evaporation‐based power generation efficiency [16]. On the other hand, a hydrophobic substrate conventionally exhibits weak affinity with water, causing water molecules to form droplets, resulting in high contact angles on the surface [17]. As a result, water gathers in spherical shapes on the hydrophobic surface, concentrating electron movement in specific areas. Particularly during water flow, the weak adhesion of water to the surface allows it to flow more rapidly, though this can lead to uneven charge transfer [18]. The use of hydrophobic substrates can generate high current bursts due to rapid evaporation and may be advantageous for electricity generation by utilizing the quick movement and flow of water.

From the perspective of nanomaterials [19], hydrovoltaic power generation can be broadly categorized into metal oxide, carbon‐based, bio‐based, and polymer materials. Each material type induces differences in interactions with water, electrical conductivity, and environmental suitability. First, metal oxide materials exhibit high electrical conductivity and durability inherent to inorganic substances [20]. This leads to high power generation efficiency and excellent stability under various environmental conditions. Carbon‐based materials, on the other hand, offer not only high electrical conductivity but also a large surface area and excellent processability, such as solution processing [13]. These characteristics enable high power generation efficiency and versatility in form and application. Bio‐based materials, composed of naturally derived biomaterials, are environmentally friendly and biodegradable [21]. Therefore, these materials are suitable for sustainable power generation and have high biocompatibility. Polymer materials feature diverse chemical structures and properties, along with notable flexibility and processability [22]. These attributes allow for the creation of flexible and lightweight devices, making them suitable for wearable technology and large‐area production. Additionally, their properties can be optimized through chemical modification.

In addition to substrates and nanomaterials, external factors such as the water source also result in different characteristics in hydrovoltaic power generation. These water sources can be categorized into wastewater, seawater, and biofluids. Wastewater primarily consists of domestic sewage and industrial effluents, containing various organic and inorganic contaminants with diverse chemical properties such as pH, salinity, and pollution levels [23]. Utilizing wastewater allows for simultaneous electricity generation and wastewater treatment, addressing the need for both. Seawater contains high salinity and various ions, making it a plentiful resource that can be used on a large scale [24]. The high salt concentration enhances ion movement, potentially leading to high electricity generation efficiency. Seawater can be harnessed as a sustainable energy resource in marine environments. In case of biofluids, including blood, urine, and sweat, they are fluids derived from biological sources [25]. The biofluids contain specific biomarkers and bio‐ions, which can be exploited for specialized electricity generation using the unique properties.

The hydrovoltaic power generation phenomenon influenced by various materials and water sources can fundamentally be applied to power sources. Furthermore, it is possible to create sensors that utilize electrical signals generated by material deformation and external environment changes [26]. Additionally, due to the environmentally friendly and non‐toxic nature of the power generation method and materials, this generation technology can be expanded to sustainable and biomedical applications such as water purification and electrical stimulation [27]. With the research history and technological advancements, in this review, we suggest guidelines for a versatile and sustainable energy source related to hydrovoltaic power generation. These guidelines have the potential to indicate future directions for hydrovoltaic power generation research regarding mechanism, material, and water source to ensure high performance and novel applications. This could lead to new future industry and a viable alternative or complementary technology to traditional renewable energy sources like solar and wind power.

## 2 Mechanism

**FIGURE 1:**
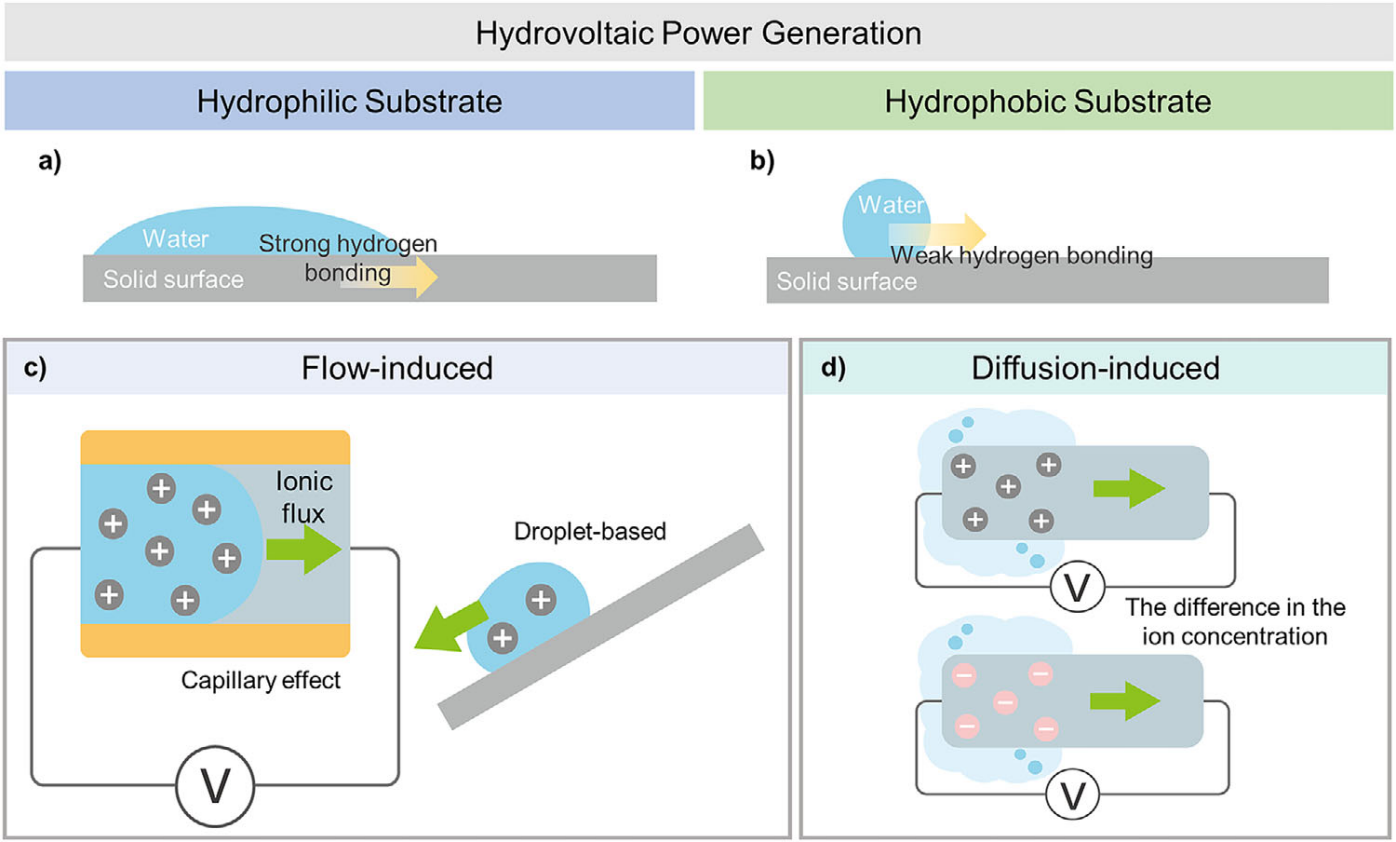
Mechanism and applications of hydrovoltaic power generation. (a,b) Schematic illustration of (a) a hydrophilic and (b) a hydrophobic substrate with water. (c,d) Schematic illustration of the working mechanism of (c) flow‐induced and (d) diffusion‐based hydrovoltaic power generation.

In hydrovoltaic power generation, the most critical factor is the reaction that occurs at the interface between water and the solid surface. Therefore, it is essential to first consider the bonding properties between water molecules and the solid surface. To understand how the energy from water is effectively transferred to the solid surface, it is necessary to grasp the interaction characteristics between water and solid surfaces, which vary according to their surface energies. The surface of a solid has different surface energies depending on its molecular characteristics, and it exhibits different wetting properties when in contact with liquids like water or oil. Typically, a solid with high surface energy allows water to wet the surface, a property known as hydrophilicity (Figure 1a,b). Conversely, a solid with low surface energy resists water wetting, a property known as hydrophobicity. These wetting properties can be measured by the contact angle of water droplets: a low contact angle indicates hydrophilicity, while a high contact angle indicates hydrophobicity.

One of the most representative indicators of wetting properties is the hydrophilic‐lipophilic balance (HLB). HLB is commonly used to indicate the balance between hydrophilicity and lipophilicity, describing how well water and oil can mix. HLB values range from 0 to 20, with lower values indicating stronger hydrophobicity and better mixing with oil, while higher values indicate stronger hydrophilicity and better mixing with water. This characteristic is determined by the chemical groups on the molecule's surface and can be calculated based on hydrophilic functional groups.

Water energy harvesting is significantly influenced by the electrical double layer (EDL) formed when water contacts a solid surface with surface charges. The EDL consists of a surface layer known as the Stern layer, where ions are adsorbed onto the solid surface, and a diffusion layer formed by counterions attracted by those ions. The EDL generates a strong electric field and creates a potential gradient, producing electrical energy. This potential, called the zeta potential, depends on the surface charge of the solid. In other words, the interaction between water and the solid is influenced by the surface energy of the solid, and this energy affects the degree of wetting. Consequently, the electrical energy induced by the EDL is affected by the surface energy differences, making it essential to understand these factors comprehensively.

In general, hydrophilic substrates form stronger hydrogen bonds with water, while hydrophobic substrates form relatively weaker bonds. Therefore, the potential energy is typically higher in hydrophilic substrates. However, from a hydrodynamic perspective, water molecules move more freely over hydrophobic substrates due to the weaker bonding between the water and the substrate. Given that water energy harvesting is affected by both the potential energy generated by EDL formation on the substrate and the movement of water from a hydrodynamic perspective, selecting an appropriate substrate depends on the specific application. We plan to address case studies focusing on increasing potential using hydrophilic substrates and enhancing water flow from a hydrodynamic perspective using hydrophobic substrates.

### 2.1 Hydrophilic Surface

Hydrophilic substrates, which bond with water molecules and form strong hydrogen bonds, are utilized in hydrovoltaic power generation (Figure 1a) [28]. The mechanism of hydrovoltaic power generation with hydrophilic substrate can be categorized into flow and diffusion (Figure 1c,d). Flow involves inducing pressure differences within the device to generate electrical power based on the movement of water [29]. In most flow‐induced hydrovoltaic power generation, water flow induced by capillary forces through narrow channels drives ionic flux within the channels, thereby inducing electrical current in the external circuit [16a, 30]. Diffusion, on the other hand, is driven by ion concentration differences within the device, which occurs through the adsorption or desorption of moisture from the air. It operates based on the phenomenon of ions diffusing from areas of high concentration to low concentration to achieve equilibrium when the ion concentration in the solvent varies. This mechanism of generating power relies on the flow of ions. In this part, hydrophilic‐based hydrovoltaic power generators are discussed depending on the mechanism of flow and diffusion with case studies.

#### 2.1.1 Flow

When water contacts with a solid surface, an electrical double layer (EDL) is formed [31]. Within the EDL, the stern layer is formed near the surface, while a diffusion layer rich in counterions is adjacent to it [32]. When water flow is induced by gravitational forces, external pressure, capillary force, etc., shearing motion occurs in the diffusion layer. In a narrow channel, the induced ionic flux by this flow of water is known as the ‘streaming current (*I*_S_)’ and can be expressed by the following equation [33].

(1) $$I_{S}=\frac{A\varepsilon_{0}\varepsilon_{r}\Delta P\zeta }{\eta r}\;$$

where *A* indicates cross‐sectional area of the nanochannels, *r* the radius of the nanochannels, ΔP the pressure difference across the nanochannel, ζ the zeta potential of the material and η the viscosity of the solution. The constants $\varepsilon_{r}$ and $\varepsilon_{0}$ indicate the permittivity of the solution and in vacuum, respectively. Additionally, the ‘streaming potential (*V*_S_)’ can be expressed by the following equation [33a].

(2) $$V_{\max}=\frac{\varepsilon_{0}\varepsilon_{r}\Delta P\zeta }{\sigma \eta }\;$$

where σ is the conductivity of the solution. The streaming current and potential are related to the water flow by the pressure difference and the morphology of the channel. In general, nanogenerator devices at a small scale utilize capillary force for generating water flow. In this regard, hydrophilic substrates have the potential to easily induce water flow due to the strong bond between the substrate and water molecules.

**FIGURE 2:**
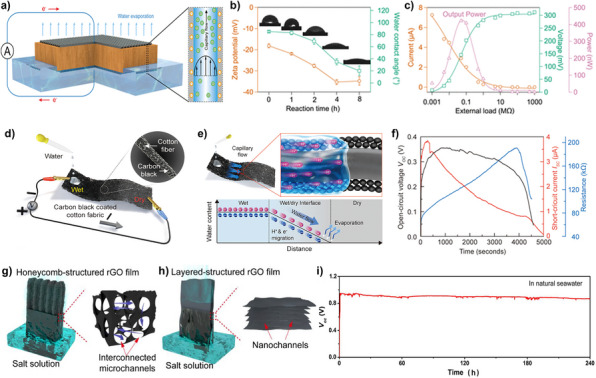
Flow‐induced power generators with hydrophilic substrates. (a–c) Anisotropic three‐dimensional wood structure‐based electricity generator. (a) Schematic illustration of electricity generation induced by evaporation in a piece of wood. (b) Comparison of the zeta potential and water contact angle from various citric acid (CA) modification durations. (c) Measured voltage and current profile with the power output of the wood structure‐based electricity generator. Reproduced with permission [33a]. Copyright 2020, American Chemical Society. (d–f) Transpiration‐driven electrokinetic power generator (TEPG). (d) Schematic illustration of the TEPG device structure. (e) Schematic illustration of the operation mechanism of a carbon‐black coated cotton fabric TEPG device. (f) Measured open‐circuit voltage (*V*_OC_), short‐circuit current densities (*J*_SC_), and resistance profiles from the TEPG (resistance of 66 kΩ) by dropping 0.25 mL of water on negative electrode. Reproduced with permission [13]. Copyright 2019, American Chemical Society. (g–i) Printed honeycomb‐structured reduced graphene oxide (rGO) film‐based electricity generator for high‐concentration salt operation. (g) Schematic illustration of the honeycomb‐structured rGO film electricity generator with interconnected microchannels. (h) Schematic illustration of the layered‐structured rGO film electricity generator with nanochannels. (i) Long‐term operation of honeycomb‐structured rGO film electricity generator. Reproduced with permission [34]. Copyright 2021, American Chemical Society.

In 2020, Zhou et al. proposed a hydrovoltaic power generator utilizing natural wood, a natural hydrophilic material, to induce capillary force for streaming potential/current generation (Figure 2a) [33a]. Natural wood possesses an intrinsic anisotropic three‐dimensional continuous microchannel structure, resulting in relatively low hydrodynamic resistance while capable of inducing high streaming current. For measuring streaming potential, a sandwich structure is fabricated with electrodes placed above and below a porous material capable of inducing water flow, such as two PET meshes coated with a conductive carbon paste (PET‐C). The wood chosen in this study primarily consists of cellulose, hemicellulose, and lignin, which are rich in hydroxyl groups, facilitating better hydrolysis as water passes through the wood microchannels. This property enables the formation of an electrical potential difference based on the pressure difference between the top and bottom surfaces. Specifically, when water is absorbed from below, natural evaporation from the top induces continuous direct current flow from capillary force‐driven water flow. An important aspect of the study is the focus on enhancing the hydrophilic property by examining the zeta potential and water contact angle, depending on the type of wood and citric acid (CA) treatment (Figure 2b). Zeta potential quantifies the potential difference between the fluid and particles within the electrical double layer (EDL) that forms at the interface between water and a charged surface. Hydrophilic surfaces typically exhibit high zeta potential, attributed to the presence of abundant surface charges that promote strong interactions with water. In contrast, hydrophobic surfaces generally display a lower zeta potential due to fewer surface charges and reduced interaction with water. Both wettability and zeta potential are closely governed by surface charge and are interrelated. In hydrovoltaic power generation, which operates based on the potential difference arising from the formation of the EDL, surfaces with a higher surface potential demonstrate stronger interactions with water, resulting in lower contact angles and, consequently, higher output voltages. Enhancing the hydrophilic property of wood promotes water infiltration through the wood channels, thereby influencing streaming potential and current. As demonstrated by the 24‐h measurements, wood devices exhibit long‐term stability in electricity output, showing resilience to changes in the surrounding environment. Moreover, when an external load of 0.1 MΩ was applied to the circuit, the wood nanogenerator exhibited a maximum power output of 450 nW (Figure 2c). Overall, the study on wood nanogenerators based on the streaming potential mechanism derived from natural evaporation demonstrates the potential for improving performance through the adjustment of variables such as hydrophilicity and pore size of nanochannels.

In 2019, Yun et al. proposed the transpiration‐driven electrokinetic power generator (TEPG) (Figure 2d) [13]. The TEPG utilizes a hydrophilic channel to induce transpiration and generate streaming potential. The TEPG device is fabricated by dipping cotton fabric into an aqueous dispersion containing carbon nanoparticles mixed with sodium dodecylbenzene sulfonate (SDBS) surfactants using a dip coating process. After dip coating, the TEPG is completely dried and ready for use as a hydrovoltaic power generator. The TEPG operates when an asymmetrically wetting state is induced on one side of the device by dropping water droplets. An electrical double layer (EDL) forms on the surface of carbon particles when the water droplet falls, creating a potential difference due to EDL formation on only one side, thus generating DC voltage (Figure 2e). Due to the hydrophilic nature of the cotton fabric, water capillary flow is induced from the wet to the dry side. Protons dissolved in the water flow in the direction of water flow, and electrons in the carbon particles also flow in the same direction due to electrostatic attraction between protons and electrons. In addition, the resistivity increases due to hydration over time, leading to a decrease in current. The initial resistance of TEPG varies depending on the concentration of carbon black coated on the cotton fabric, and the electrical performance of TEPG can be optimized by adjusting this resistance. The TEPG demonstrates DC output for over 4000 s, with a maximum of 0.53 V and 3.91 µA (Figure 2f).

In 2021, Wu et al. proposed the printed honeycomb‐structured reduced graphene oxide (rGO) film‐based electricity generator (Figure 2g,h) [34]. Building upon the mechanism of streaming potential generated by water molecules flowing through active materials under capillary forces, the influence of surface area and ions for enhancing streaming potential is investigated. To achieve this, honeycomb structures and layered structures of rGO film are selected (Figure 2g,h). The honeycomb‐structured film with interconnected microchannels is fabricated using 3D printing technology, employing a freezing‐casting process with ice crystals as templates. Meanwhile, the layer‐structured rGO film with abundant nanochannels is also fabricated using the same 3D printing technology but without the freezing‐casting process. Electrodes are formed on the rGO film by coating CNT paste on both sides and connecting copper wire on them, followed by encapsulation with epoxy to create the final device. The device is partially immersed in a solution to induce water flow via capillary force and demonstrate applicability to natural water, consistently delivering a stable open‐circuit voltage (*V*_OC_) of approximately 0.83 V with a power density of 0.79 µW cm^−2^ in seawater for over 240 h, day and night (Figure 2i). Notably, the presence of hydrophilic groups on the honeycomb‐structured film improved water uptake, facilitating effective capillary action along the microchannels. When comparing the surface areas of the fabricated films, the honeycomb‐structured film exhibited a specific surface area sevenfold larger than that of the layer‐structured film. Due to the electrokinetic effect, a higher density of exposed surface charge leads to a greater participation of counterions in forming streaming potential. Consequently, the honeycomb‐structured rGO film, possessing more surface charge, exhibited a higher measured *V*_OC_ compared to layered‐structured rGO film.

#### 2.1.2 Diffusion

When a local or global ion gradient occurs in a specific aqueous solution, the gradient dissipates and reaches equilibrium spontaneously [35]. However, continuous ionic transport is possible if the ion gradient is artificially maintained. Many researchers have introduced material modifications, such as functional groups or hygroscopic materials, to artificially maintain the ion gradient in water energy harvesting devices. Hygroscopic materials like hydrogels and composite aerogels have been introduced to maintain the ion gradient, converting chemical potential into electrical power. In other words, by altering functional groups on the material or asymmetrically incorporating hygroscopic materials, the ion gradient can be artificially sustained, enabling continuous diffusion‐based output voltage. In this regard, by utilizing the mechanism of adsorption or desorption of moisture in the air, an ion gradient can be induced through the spontaneous adsorption of water on a solid surface. Oxygen‐functional groups primarily play a critical role in spontaneously adsorbing moisture from the air, and this asymmetric structure of functional group concentration induces the diffusion of ions. Therefore, hydrovoltaic power generation occurs when we induce an ion gradient within a device, causing ions to flow along the concentration gradient and induce a steady ionic current (Figure 1d). The ion gradient induces charge transfer within the system, forming an electrical potential at each end of the device, which results in current flow through an external circuit.

**FIGURE 3:**
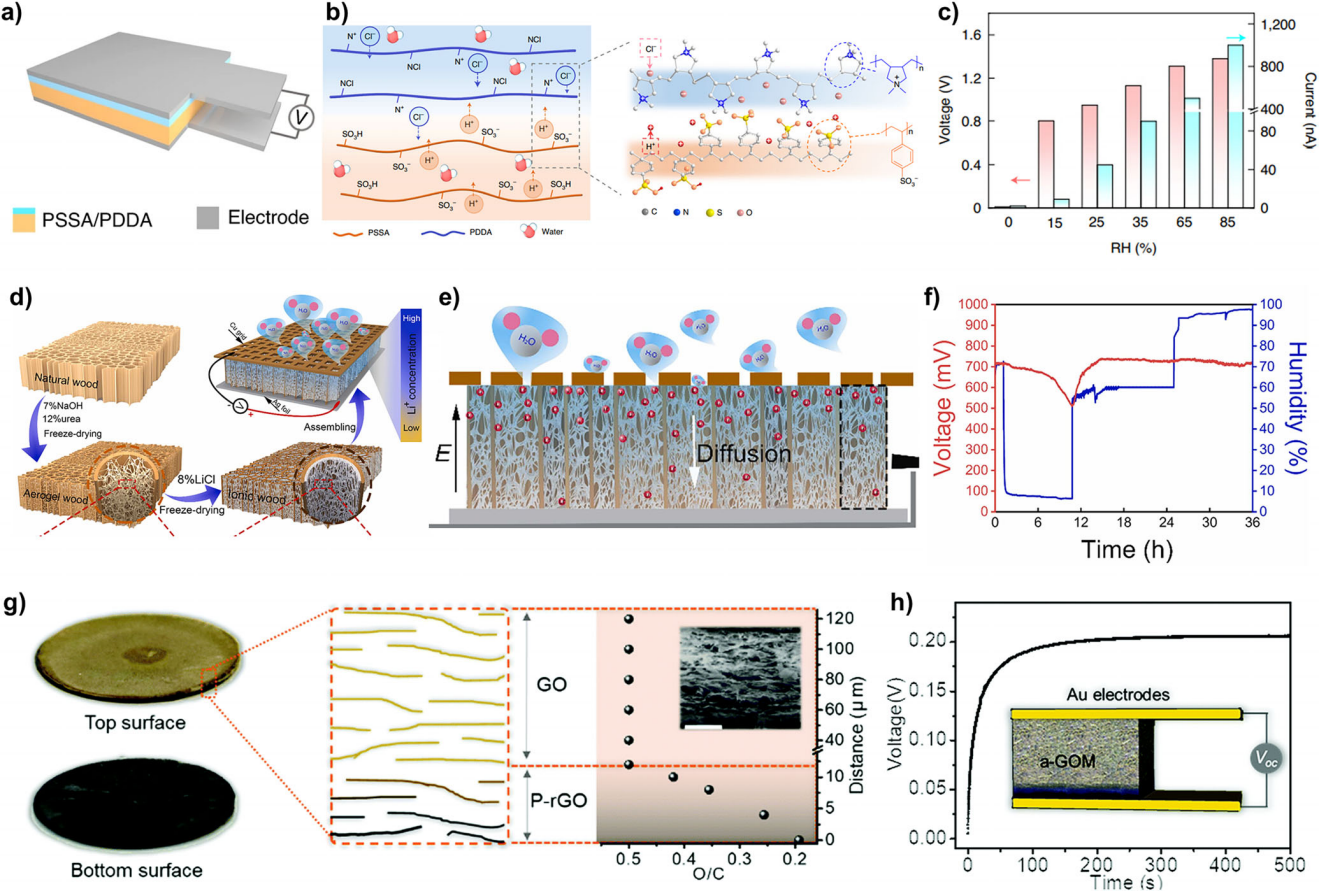
Diffusion‐based power generators with hydrophilic substrate. (a–c) A heterogeneous moisture‐enabled electric generation (HEMG) based on a bilayer of polyelectrolyte films. (a) Schematic illustration for testing HMEG's electrical performance. (b) Surface potential at the inner leaflet (IL) is vastly different from that at the outer leaflet (OL), and the difference between the two surface potentials. (c) Voltage and current output in response to variation in RH (at 25°C). Reproduced with permission [14]. Copyright 2021, Springer Nature. (d–f) A hydrovoltaic generator made of ionic wood with continuous nanostructures with LiCl salts around the inner walls of wood microchannels. (d) Schematic illustration of preparation process of ionic wood and assembling of the generator. (e) Schematic diagram of moisture‐dependent power generation of the device. (f) Comparison of the output voltage and humidity with real‐time humidity change. Reproduced with permission [36]. Copyright 2022, Elsevier. (g,h) A spontaneous electric generation with asymmetric porous graphene oxide membrane (a‐GOM). (g) Schematic diagram of a‐GOM that is composed of upper GO with homogeneous structure and bottom P‐rGO with gradient oxygen‐containing groups. (h) The *V*_OC_ of a‐GOM between two Au electrodes under ambient conditions with relative humidity (RH) of ≈25% at room temperature of about 20°C. Reproduced with permission [37]. Copyright 2018, The Royal Society of Chemistry.

In 2021, Wang et al. proposed a heterogeneous moisture‐enabled electric generator (HMEG) based on a bilayer of polyelectrolyte films (Figure 3a) [14]. The HMEG generates power by inducing the diffusion of oppositely charged ions through the spontaneous adsorption of water molecules from the air. A biomimetic bilayer of polyelectrolyte films (BPF) is constructed by combining polycation (polydiallyl‐dimethyl‐ammonium‐chloride) (PDDA) and polyanion (polystyrene sulfonic acid (PSS) and polyvinyl alcohol (PVA) hybrid film (PSSA)) films (Figure 3b). The HMEG, with its bilayer of polyelectrolyte films (BPFs) incorporating a heterogeneous distribution of charged mobile ions (Cl^−^ and H^+^), generates 0.95 V at 25% RH, increasing to 1.38 V at 85% RH (Figure 3c). The rational introduction of PVA components endows the PSSA film with superior flexibility. The BPF can spontaneously adsorb water molecules from moist air, with negatively charged Cl^−^ ions dissociated from the PDDA layer and positively charged H^+^ ions from the PSSA layer. The superior hydrophilicity of BPF enables high performance through diffusion‐based hydrovoltaic power generation. The ion dissociation process, combined with continuous adsorption of water molecules in the HMEG, allows mobile ions to overcome the inner electric field, enabling constant diffusion over the long term. Therefore, continuous water adsorption and induced diffusion of oppositely charged ions generate current flow and voltage output. Additionally, the researchers introduce a sequentially aligned stacking (SAS) strategy to create a large‐scale HMEG unit. By connecting 1600 HMEG units in series, a very high voltage of 1000 V is achieved at 25% RH, significantly contributing to the advancement of hydrovoltaic power generation and demonstrating its scalability.

In 2022, Li et al. proposed an ionic wood processed from natural wood‐based spontaneous electricity generator (Figure 3d) [36]. They developed an effective strategy to form continuous nanostructures with LiCl salts resembling spider webs within natural wood for the generator. An aerogel wood with intact top–down wood microchannels was modulated with ion providers (LiCl) similar to spiderwebs, providing sufficient mobile ions (Li^+^) and continuous ionic bridges for moisture‐induced power generation. The wood for the generator underwent treatment through a freeze‐drying process to produce aerogel wood, which was then immersed in an 8 wt% LiCl solution to establish sufficient ion networks on the cellulose nanostructures (Figure 3d). The specific spider‐web‐like structure and ion provider of LiCl enhance the wood's hygroscopic and water‐holding abilities, resulting in high electricity generation. The top side of the generator is exposed to air, capturing moisture and releasing free Li^+^ ions, while the underside is sealed by Ag foil, creating a reverse condition (Figure 3e). This setup establishes a humidity gradient and facilitates ion diffusion from bottom to top, leading to the spontaneous generation of 750 mV and 712 µA. The generator also demonstrates prolonged stability during sudden humidity changes, showcasing an energy storage effect and promising future applications (Figure 3f).

In 2018, Cheng et al. proposed a spontaneous electricity generator with a graphene oxide membrane (GOM) prepared using a directionally‐induced thermal reduction strategy (Figure 3g) [37]. This asymmetric porous graphene oxide membrane (a‐GOM) consists of two parts: A partially thermally reduced GO (P‐rGO) layer with gradient oxygen‐containing groups and the original GO layer with a homogeneous structure. Based on this asymmetric structure, sufficient protons in the GO layer spontaneously moved to the side with fewer oxygen‐containing groups, resulting in internal charge separation. Figure 3g illustrates the prepared a‐GOM, where the top surface is the original brown, and the bottom side has converted from the original brown GO to black‐gray P‐rGO through thermal treatment. With this directional thermal treatment process, the successful regulation of oxygen‐containing groups in the GO assembly and the construction of an asymmetric structure in a‐GOM were achieved. The strong absorption capacity of a‐GOM for water molecules from the air causes positively charged protons and negatively charged oxygenated functional groups (GO^−^) to dissociate in pairs within a‐GOM, resulting in spontaneous diffusion. Therefore, the charge separation within a‐GOM between the GO and P‐rGO parts induces a built‐in potential with proton diffusion, resulting in an electrical output of 150–450 mV. The maximum output power of the generator is about 18.4 nW cm^−2^, as measured by the voltage and current with different electric resistors (Figure 3h).

### 2.2 Hydrophobic Surface

The phenomenon of energy generation via hydrophobic materials stems from surface electrification [38], a process wherein hydrophobic materials interact with water molecules. Upon contact, the negatively charged hydrophobic surface induces the attraction of cations present in the water droplet, leading to the formation of an electrical double layer at the interface [31]. Subsequent movement of water droplets initiates the migration of charges along the hydrophobic surface‐droplet interface, facilitating the dissipation of charge imbalances, which generates electrical current. While hydrophilic surface induces water droplet movement through the attraction of water molecules, hydrophobic materials lack an inherent driving force for moving water droplets. Consequently, the movement of water droplets on hydrophobic surfaces depends on external driving forces, such as the inclination of devices or the introduction of moving water droplets. This dynamic process results in the hydrovoltaic energy generation.

#### 2.2.1 Flow

**FIGURE 4:**
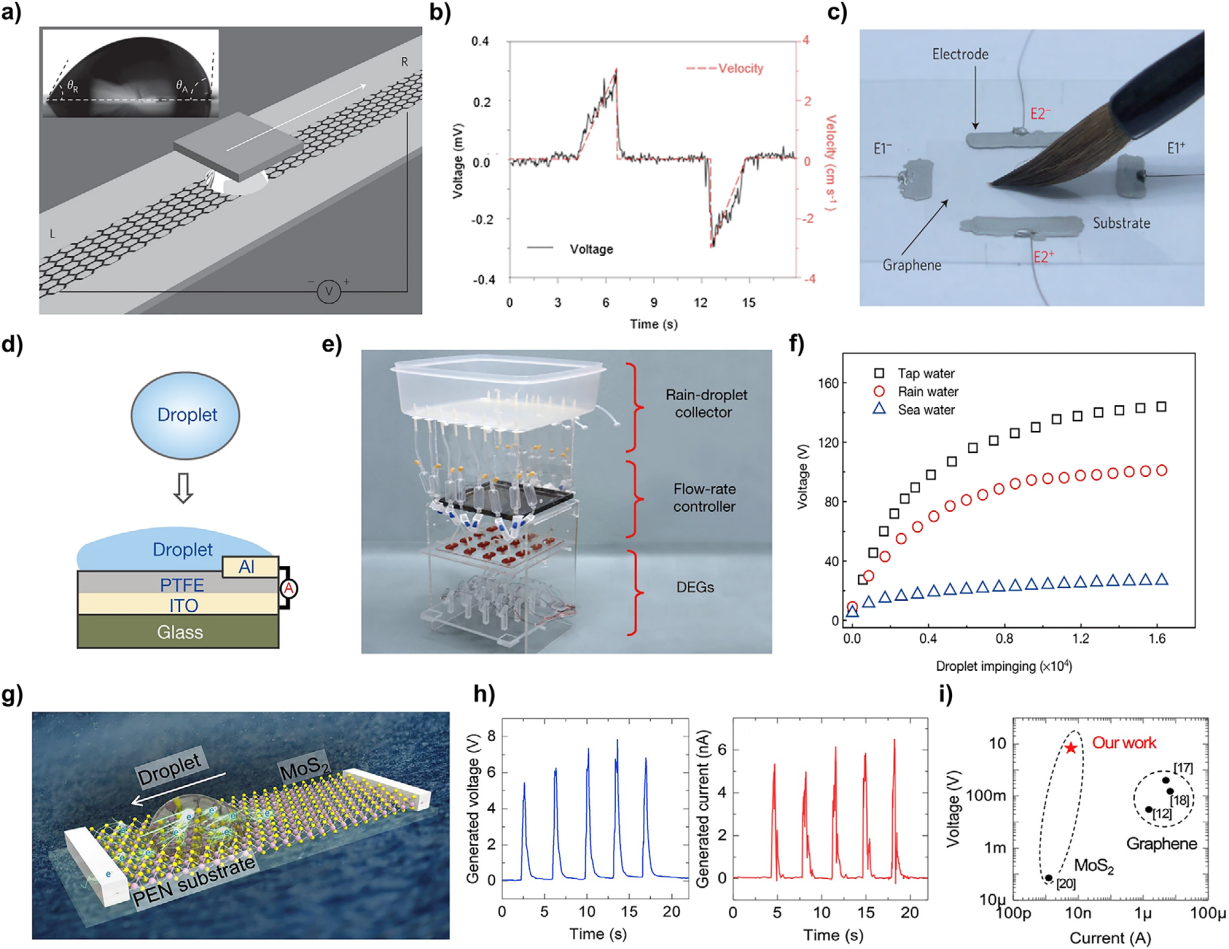
Flow‐induced power generators with hydrophobic substrate. (a–c) Graphene‐based hydrovoltaic power generator. (a) Schematic illustration of the graphene‐based energy harvester utilizing a moving ionic liquid. (b) Correlation between voltage and ionic liquid droplet velocity. (c) Photograph of the graphene‐based hydrovoltaic power generator. Reproduced with permission [39]. Copyright 2014, Springer Nature. (d–f) Hydrovoltaic power generator employing PTFE. (d) Schematic diagram of the PTFE. (e) Optical image of the fabricated raindrop collecting and dispensing system. (f) Voltage performances with tap water, raindrop and seawater. Reproduced with permission [40]. Copyright 2020, Springer Nature. (g–i) MoS_2_‐based hydrovoltaic power generator. (g) Schematic illustration of the MoS_2_‐based hydrovoltaic power generator. (h) Voltage and current characterization of the MoS_2_‐based hydrovoltaic power generator. (i) Performance comparison of the MoS_2_ power generator with other hydrovoltaic power generators. Reproduced with permission [18]. Copyright 2020, Elsevier.

Jun et al. conducted experiments to investigate the concept of hydrovoltaic generation originating from hydrophobic materials [39]. Specifically, monolayers of graphene were synthesized via chemical vapor deposition (CVD) and subsequently transferred onto a polyethylene terephthalate (PET) substrate. Following the fabrication of graphene samples, a droplet of 0.6 M sodium chloride (NaCl) was sandwiched between a wafer and the graphene layer to facilitate manipulation of droplet movement. This setup (illustrated in Figure 4a) was designed to enable precise control of droplet movement across the graphene surface. This investigation revealed a direct relationship between the velocity of the droplet and the voltage associated with the generated hydrovoltaic energy from graphene (Figure 4b). Furthermore, Jun et al. proposed that the radius of ions exhibits an inverse proportionality to the voltage generation, as evidenced by experiments involving several salts. Lastly, Jun et al. quantified the drawing potential through manual handwriting using a Chinese brush in conjunction with the graphene layer (Figure 4c).

Xu et al. developed a droplet‐based electricity generator by introducing polytetrafluoroethylene (PTFE) onto indium tin oxide (ITO) glass [40]. The PTFE precursor was drop‐cast onto the ITO glass and subsequently heated to form a PTFE film. An aluminum electrode was then attached to the ITO glass (Figure 4d). The high charge accumulation characterization of PTFE was aimed to be applied in storing electrical charges from droplets. With this PTFE—ITO hydrovoltaic power generator, Xu et al. fabricated a raindrop collector‐connected hydrovoltaic power generator (Figure 4e). The device demonstrated an output of approximately 150 V and 300 µA. Furthermore, the correlation between electrical output and various parameters was investigated, including droplet volume, type of water, and the number of impinging droplets. These findings revealed that increasing the droplet volume led to an increase in charge, and that tap water generated a higher voltage than seawater and rainwater (Figure 4f).

Adha et al. proposed an hydrovoltaic energy harvesting system employing molybdenum disulfide (MoS_2_) deposited on a polyethylene naphthalene (PEN) substrate (Figure 4g) [18]. Initially, MoS_2_ was synthesized on a polished c‐plane sapphire substrate utilizing the CVD method. Subsequently, the MoS_2_/sapphire composite was transferred onto the PEN film utilizing a surface‐energy assisted transfer technique. Upon contact with a 0.6 M NaCl droplet, the MoS_2_ device did not exhibit electricity generation. However, subsequent tilting of the sample induces water movement, resulted in the generation of electricity by the hydrovoltaic effect. Voltage and current characteristics of MoS_2_ hydrovoltaic power generator were measured, demonstrated the capability to generate about 8 V when subjected to a moving NaCl droplet (Figure 4h). Also, performance comparison shows that MoS_2_ hydrovoltaic power generator has high electrical potential compared to other hydrovoltaic power generators (Figure 4i).

#### 2.2.2 Diffusion

The operational principles underlying diffusion‐based water energy harvesting via hydrophobic surfaces share similarities with hydrophilic hydrovoltaic power generators, although there are notable differences in their modes of operation. While certain hydrovoltaic power generators may exhibit static conditions of water droplets due to their hydrophobic nature, necessitating the inclination or the movement of the harvester to induce water movement. However, diffusion can serve as a driving force for water movement in select instances [41]. On hydrophobic surfaces, the driving force of diffusion can be either an ionic gradient or a water concentration gradient induced by evaporation. This has led to the research of capillary channels as a potential candidate for diffusion‐based hydrophobic hydrokinetic power generators.

**FIGURE 5:**
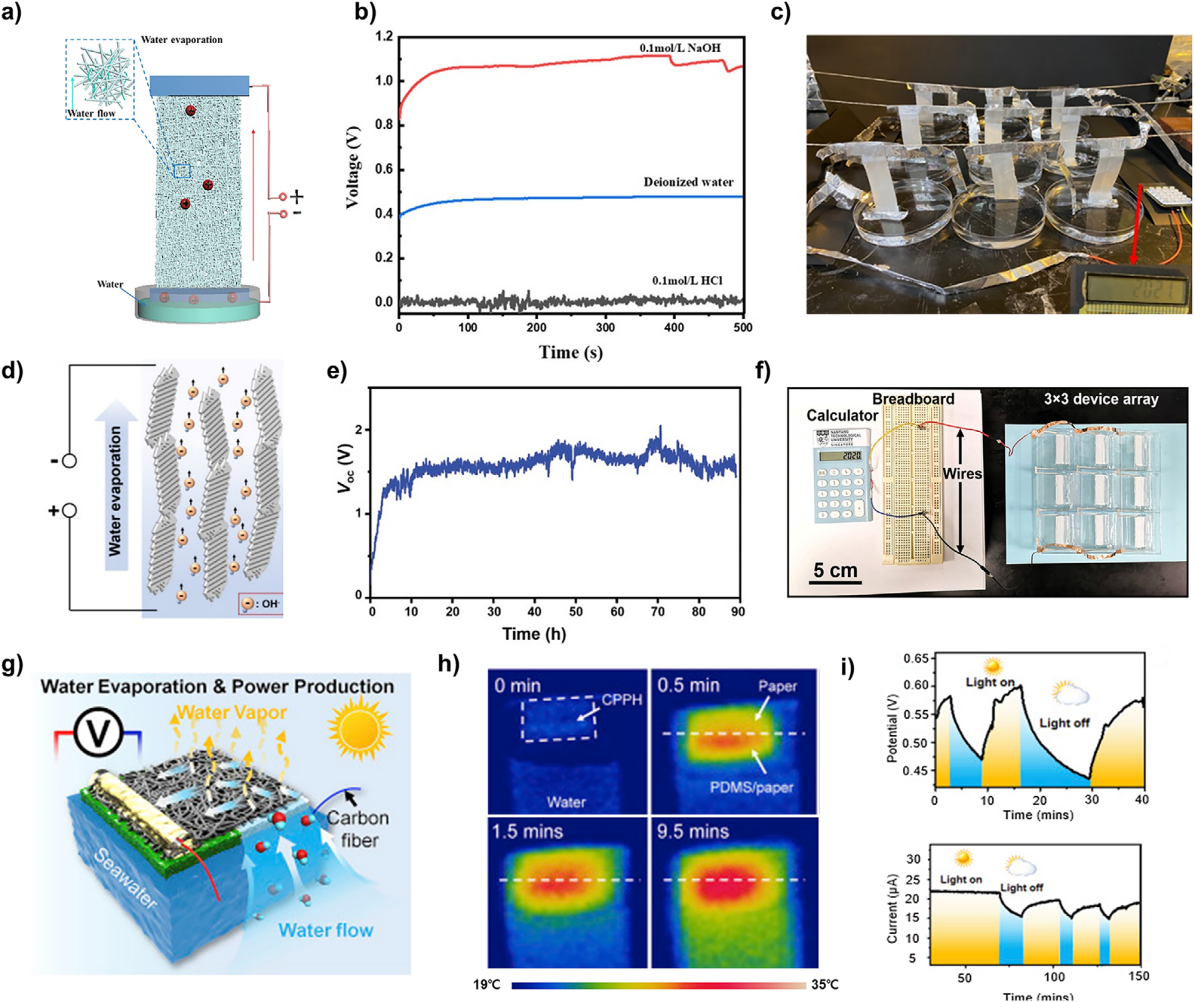
Diffusion‐based power generators with hydrophobic substrate. (a–c) Ceramic nanofiber‐based power generator. (a) Schematic illustration of SiO_2_ nanofiber‐based power generator. (b) Voltage performance difference between DI water, 0.1 M HCl and 0.1 M NaOH solution. (c) Photograph of power generator devices connected to a calculator. Reproduced with permission [42]. Copyright 2021, American Chemical Society. (d–f) MOF‐utilized power generator. (d) Schematic diagram of the AlOOH/UIO‐66 power generator. (e) Long‐term open circuit voltage performance of AlOOH/UIO‐66 power generator. (f) Photograph image of 3 × 3 device array of AlOOH/UIO‐66 power generators connected with calculator. Reproduced with permission [43]. Copyright 2020, Wiley‐VCH GmbH. (g–i) Carbon nanotubes (CNTs)/PDMS/paper hybrid (CPPH) power generator. (g) Schematic illustration CNTs‐based asymmetrical power generator. (h) IR images showing the temperature change of CNTs under 1 sun illumination. (i) Voltage and current characterization of CPPH under light on and off status. Reproduced with permission [44]. Copyright 2020, Elsevier.

Zhaoyang et al. proposed a SiO_2_ nanofiber hydrovoltaic power generator fabricated through sol‐gel electrospinning (Figure 5a) [42]. The nanochannels inherent in the SiO_2_ structure, coupled with evaporation at the top portion, drive water flow and subsequently generate electricity. The SiO_2_ nanofibers were synthesized using the sol–gel electrospinning technique, employing tetraethyl orthosilicate (TEOs) as the precursor material. After fabricating the SiO_2_ nanofibers, aluminum foils were attached as electrodes. The electrical performance of the device was evaluated under various conditions, including membrane thickness, fiber diameter, coverage state, and exposure to acidic or basic solutions (Figure 5b). Results indicated a proportional relationship between device thickness and output voltage, with uncovered devices outperforming covered ones. Notably, the device exhibited higher voltage generation in basic solutions compared to deionized water, while voltage generation in acidic solutions approached zero. This phenomenon is attributed to the preferential adsorption of hydronium ions at basic sites and hydroxide ions at acidic sites, creating an ionic gradient facilitated by aluminum's adsorption of positive charges in water, thereby enhancing the device's performance (Figure 5c).

Qinglang et al. synthesized a metal‐organic framework (MOF) known as UIO‐66, employing a method involving the growth of UIO‐66 on 2D aluminum oxyhydroxide (AlOOH) nanoflakes (Figure 5d) [43]. Leveraging the high porosity inherent in MOFs, the facilitated transport of water molecules via capillary action was observed. This process, coupled with water evaporation, engenders an asymmetrical wetness gradient within the energy harvester, consequently resulting in continuous energy generation (Figure 5e). To ascertain the influence of evaporation on power generation, open circuit voltage measurements were conducted under both covered and uncovered conditions. Remarkably, a discernible voltage drop was noted when the samples were covered, indicative of the impact of evaporation on the energy generation mechanism. This experimental validation highlights the significant role of capillary force facilitated by the porous structure of MOFs, and the sustained operation driven by evaporation (Figure 5f).

Xiao et al. proposed a CNTs‐based evaporation energy harvester. A CNTs film was fabricated on paper using a spray‐coating method and passivated it with polydimethylsiloxane (PDMS) to prevent direct wetting (Figure 5g) [44]. Due to the hydrophobic properties and low surface tension of PDMS, the fabricated device floats on water. The uncovered region of the paper absorbs water and delivers it to the passivated region by diffusion. The CNTs induce water evaporation through a photothermal effect, maintaining asymmetrical wetting. Surface temperature measurements under one sun illumination confirmed the photothermal effects, verifying that CNTs generate heat for evaporation when illuminated (Figure 5h). Mass change measurements showed an increasing rate corresponding to the intensity of illumination. The voltage and current of the CNTs/PDMS/paper hybrid (CPPH) were also measured, with the CPPH achieving approximately 0.6 V and 22 µA under illumination. Moreover, the activation of a droplet sensor has been achieved using the CPPH, demonstrating an application of this device (Figure 5i).

## 3 Materials

### 3.1 Metal Oxides

In general, metal oxides are considered as a hydrophilic material [53]. Due to its hydrophilicity and robustness characterization of metal oxides, various metal oxides such as titanium dioxide (TiO_2_) [45, 54] and aluminum oxide (Al_2_O_3_) [1, 46] can be a candidates for metal oxide‐based hydrovoltaic power generators. Metal oxide hydrovoltaic power generators tend to exhibit a high output voltage of over 1 V per device. Also, metal oxides hydrovoltaic power generators have a long duration time due to their robustness.

**FIGURE 6:**
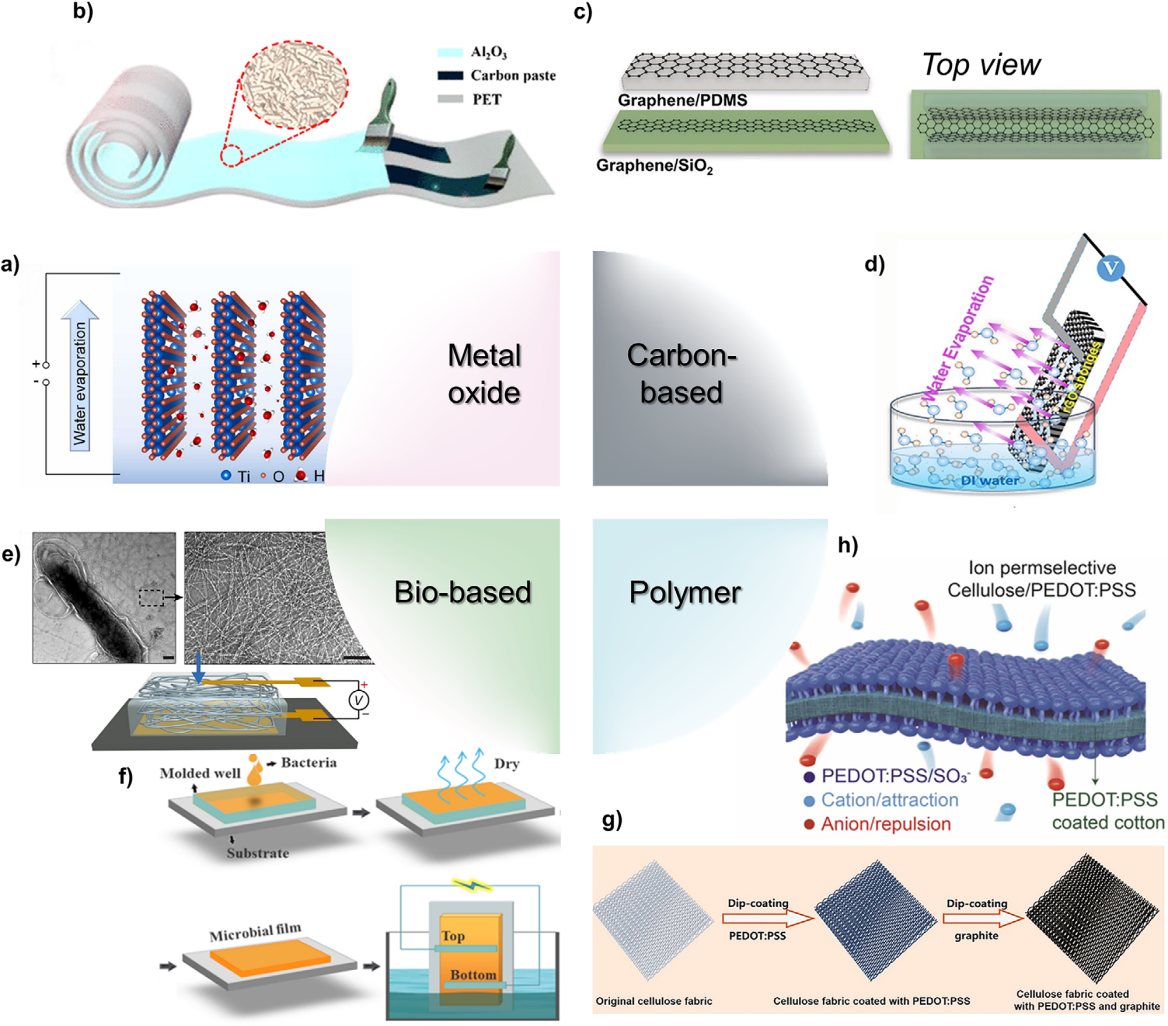
Hydrovoltaic power generator under materials classification. (a) TiO_2_‐based hydrovoltaic power generator. Reproduced with permission [45]. Copyright 2023, Elsevier. (b) Al_2_O_3_ based hydrokinetic power generator. Reproduced with permission [46]. Copyright 2019, American Chemical Society. (c) Graphene based hydrovoltaic power generator. Reproduced with permission [47]. Copyright 2022, American Chemical Society. (d) Graphene oxide (GO) sponge hydrovoltaic power generator. Reproduced with permission [48]. Copyright 2019, Elsevier. (e) Hydrokinetic power generator using protein nanowires. Reproduced with permission [49]. Copyright 2020, Springer Nature. (f) *Geobacter sulfurreducens* biofilm hydrokinetic power generator. Reproduced with permission [50]. Copyright 2022, The American Association for the Advancement of Science. (g) Hydrovoltaic power generator using PEDOT:PSS. Reproduced with permission [51]. Copyright 2021, American Chemical Society. (h) TEPG utilizing ion‐permselective characterization of PEDOT:PSS. Reproduced with permission [52]. Copyright 2022, Elsevier.

In Figure 6a, a TiO_2_‐based hydrovoltaic power generator is introduced [45]. Liu et al. fabricated cation‐deficient Ti_1‐δ_O_2_ nanosheets to enhance hydrophilicity. To investigate this, voltage measurements were conducted with varying Ti vacancy levels. It was observed that as the Ti vacancy increased, the output voltage also increased. Additionally, the voltage was analyzed under various temperature and relative humidity conditions to demonstrate that the device operates based on water evaporation. The results showed a proportional relationship between temperature and output voltage, while relative humidity and electrical performance exhibited an inverse relationship. Furthermore, long‐term measurements indicated a consistent generation of approximately 1.5 V over a period of 250 h.

In Figure 6b, an Al_2_O_3_‐based hydrovoltaic power generator is fabricated [46]. By depositing Al_2_O_3_ onto a polyethylene terephthalate film, a flexible hydrovoltaic power generator was fabricated. The electrical performance of the device was evaluated in various forms, including cylindrical, folded, and conical shapes. The voltage and current remained unaffected by the film's shape, consistently generating over 2 V. Additionally, a stretchable pattern was created using laser patterning, which did not impact the device's electrical performance. Long‐term measurements over a period of 10 days demonstrated the stability of these devices. Several studies suggest that the structure of hydrovoltaic devices does not significantly affect electrical power generation performance. However, the author argues that the structure of hydrovoltaic devices can indeed influence performance. According to the electrokinetic mechanism, power characteristics improve when counterions exhibit a structure that facilitates their mobility rather than remaining stationary. Thus, some articles propose that capillary‐inducing structures can enhance electrical power generation [33, 34, 43].

### 3.2 Carbon‐Based

Carbon is readily obtainable and abundantly available on Earth. Carbon‐based materials possess several advantages, including high conductivity and a large surface area. From these properties, carbon‐based materials, such as graphene [39, 55] and carbon black [13], are frequently utilized in hydrovoltaic power generators. When combined with hydrophilic materials, carbon materials result in highly hydrophilic and conductive hydrovoltaic power generators capable of producing high short‐circuit current densities (*J*_sc_).

In Figure 6c, Kong et al. proposed a hydrovoltaic power generator utilizing graphene, a carbon‐based material [47]. A graphene layer was deposited onto PDMS and SiO_2_ layers through a transfer process. Two graphene/PDMS layers are positioned perpendicularly to the graphene/SiO_2_ layer, forming a graphene microfluidic channel. This microfluidic channel enhances fluidic flow, resulting in hydrovoltaic power generation. This device can generate approximately 0.15 V and 2.41 µA using a 0.6 M NaCl solution. Furthermore, the voltage performance of the device is influenced by the velocity of water and applied pressure, indicating its potential for use in sensors for bending and pressure measurements.

Figure 6d presents a schematic illustration of a rGO sponge‐based hydrovoltaic power generator [48]. The rGO, characterized by a higher surface area as supported by Brunauer–Emmett–Teller (BET) analysis, enhances water kinetics, thereby improving the performance of the hydrovoltaic power generator. Moreover, Zhang et al. introduced UV and O_3_ treatments to enhance the hydrophilicity of the device. Contact angle measurements were conducted, revealing that untreated rGO exhibited an angle of 18°, whereas UV and O3‐treated rGO facilitated water droplet permeation. Furthermore, the electrical performance demonstrated an approximate output of 50 µA, markedly surpassing that of inorganic materials.

### 3.3 Bio‐Based

In the context of sustainable development, green energy has emerged as a crucial area of research. Bio‐based materials, configured with eco‐friendly components such as proteins to mimic biological entities, offer a less polluting alternative to petroleum‐based materials. Furthermore, hydrovoltaic power generation, which produces electrical power using water, a resource abundantly available on Earth, presents a promising solution for sustainable energy production.

A protein nanowire is introduced for a hydrovoltaic power generator in Figure 6e, which uses ambient humidity as a reservoir [49]. The random distribution of nanopores in protein nanowire creates a vapor gradient, enhancing the power generation of a hydrokinetic power generator. Electrical performance was measured under approximately RH 50% conditions, demonstrating stability for more than 1500 h, equivalent to over 2 months. Additionally, the current measurement proceeded in RH 0%, 35%, and 55%. Significant change of current was shown while RH changes, revealed that this device operates with ambient moisture. In general, the electrical performance of hydrovoltaic power generators is affected by environmental factors such as temperature and relative humidity. As temperature rises, moisture evaporation is induced, which in turn activates transpiration and enhances power generation characteristics. Conversely, increased humidity reduces evaporation, resulting in limited transpiration and a decline in power output. However, with the introduction of hygroscopic materials or moisture‐capturing structures, this general relationship may not hold. Therefore, it is recommended to assess the electrical performance of these devices under various environmental conditions.

A hydrovoltaic power generator utilizing *Geobacter sulfurreducens* was fabricated, as described in Figure 6f [50]. The fabricated *Geobacter sulfurreducens* film exhibited a contact angle of 59°, indicating its hydrophilic properties. This device was immersed in water, as shown in Figure 6f. Evaporation plays a crucial role in the functioning of this device; therefore, the temperature was controlled to increase the evaporation rate. Higher temperatures resulted in a higher evaporation rate, leading to enhanced electrical performance. Additionally, the research team highlighted that the microbial film offers advantages such as higher yield and simpler procedures compared to the use of protein nanowires.

### 3.4 Polymer

To augment the conductive properties of materials, conductive polymers emerge as a promising candidate. These polymers offer facile dispersal in solvents, facilitating simplified processing procedures. Moreover, polymers afford the incorporation of diverse functional groups and properties, conferring notable advantages such as ion‐permselectivity. Additionally, conductive polymers demonstrate superior conductivity in comparison to alternative materials employed in hydrovoltaic power generators, thus facilitating the generation of heightened electrical currents.

In Figure 6g, horizontal PEDOT:PSS and graphite‐coated cellulose fabric electrodes were combined with vertical silicon nanowires to facilitate both evaporation and electricity generation [51]. The hydrophilicity of silicon nanowires, resembling trees, enabled high capillary force. The surface area of the cellulose fabric was enhanced through the repeated loading of graphite, as analyzed by BET analysis. Electrical performance measurements revealed that both voltage and current density increased with elevated temperatures, in accordance with this conceptual framework. At room temperature, this device achieved an output voltage of approximately 0.5 V and a current density of 20 µA cm^−2^.

In Figure 6h, an ion‐permselective cellulose/PEDOT hydrovoltaic power generator was investigated [52]. The negative charge stemming from the sulfonate groups of PSS results in the propensity of the PEDOT membrane to interact with cations. This interaction enhances the charge density through electrostatic adsorption, in addition to the physical adsorption of water. The performance of this device varies upon its resistance, demonstrating an output voltage ranging from approximately 0.4 to 0.6 V and a current density between 6 and 13 µA cm^−3^ when utilizing deionized water (DI water). However, the utilization of electrolytes such as LiCl solution led to a significant increase in energy density. The researchers proposed that ionic solutions such as sweat and seawater have the potential to yield higher electrical performance.

A summary and comparison of the main characteristics, such as substrate, mechanism, materials, water resources, and output performance of the representative hydrovoltaic power generator are listed in Table 1.

**TABLE 1:** Summary of various hydrovoltaic power generators.

Substrate	Mechanism	Materials	Water resources	Voltage [V]	Current [A] or Current density [A cm^−2^]	Reference
Hydrophilic	Flow‐induced	rGO	DI water	0.53	1.5 µA cm^−2^	[34]
		Carbon black/Cotton fabric	DI water	0.53	3.91 µA	[13]
		Carbon black/PVA film	Seawater	1.04	165 µA	[16b]
		Natural wood	DI water	0.3	10 µA	[33a]
	Diffusion	TiO_2_	DI water	1.5	1.3 µA	[45]
		Al_2_O_3_	DI water	3	0.2 µA	[46]
		Graphene	DI water	0.15	2.41 µA	[47]
		rGO	DI water	0.44	50 µA	[48]
		GO	DI water	0.45	0.9 µA cm^−2^	[37]
		GO	DI water	1.5	136 nA	[56]
		MWCNT/Al_2_O_3_ plate	DI water	1.2	475 nA	[26]
		Carbon black	Seawater	0.43	10.05 µA	[27a]
		Carbon nanoparticle/Silicon nanowire	Biofluids	0.45	10.2 µA	[57]
		G. Sulfurreducens biofilm	DI water	0.53	2.28 µA	[50]
		Protein nanowire	DI water	0.53	250 nA	[49]
		Ionic wood	DI water	0.75	712 µA	[36]
		Polyelectrolyte film/PDDA/PSSA	DI water	1.38	5.52 µA cm^−2^	[14]
		PEDOT:PSS	DI water	0.55	22 µA cm^−2^	[51]
		PEDOT:PSS	DI water	0.58	4.2 µA cm^−3^	[52]
Hydrophobic	Flow‐induced	MoS_2_	DI water	5	5 nA	[18]
		Graphene	DI water	0.0002	—	[39]
		PTFE	DI water	143.5	270 µA	[40]
		PEDOT:PSS/CNTs/Nanofiltration membrane	Wastewater	0.24	82.3 µA	[58]
	Diffusion	SiO_2_	DI water	0.5	0.35 µA	[42]
		CNT	Seawater	0.6	22 µA	[44]
		AlOOH	DI water	1.63	0.6 µA	[43]

## 4 Water Resources

Water sources from wastewater [58], seawater [16b], and biofluids [57] are also important factors for different applications and characteristics in hydrovoltaic power generation. According to the mechanism of hydrovoltaic power generation, ions in the aqueous solution are the main factors determining the power of the generator, inducing a flow of continuous current and voltage with a potential difference within the device. In this context, these water sources can be categorized into wastewater, seawater, and biofluids. Each resource has powerful potential that can influence environmental problems and broaden the applications of hydrovoltaic power generation if these resources are utilized.

**FIGURE 7:**
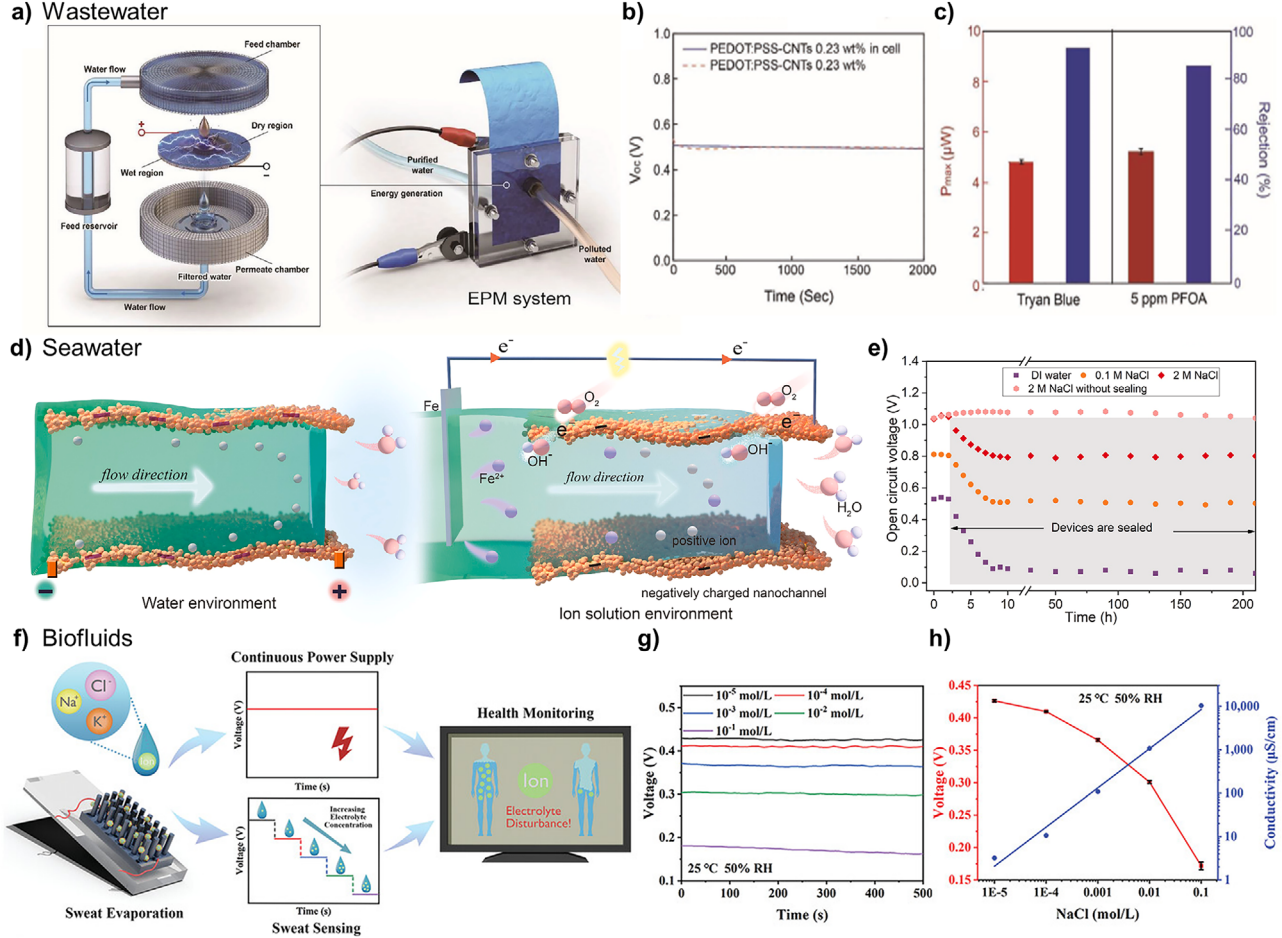
Various resources for hydrovoltaic power generation. (a–c) Electricity generation and purification membrane (EPM) with a bidirectional water stream. (a) Schematic illustration of the EPM system and its operation principle. (b) Poly (ethylene oxide) rejection by EPM with different types of support membranes. (c) Single salt (0.1 m of Na_2_SO_4_, MgSO_4_, CaCl_2_) and PFOA rejection by EPM on NF support. Reproduced with permission [58]. Copyright 2022, Wiley‐VCH GmbH. (d,e) A flexible dual‐mode electricity nanogenerator (DM‐ENG) for a wide dynamic range of aqueous salinity. (d) Schematic illustration of the working mechanism of the flexible DM‐ENG. (e) Voltage of the power nanogenerators with different films. Reproduced with permission [16b]. Copyright 2021, Elsevier. (f–h) An electric nanogenerator comprising modified carbon nanoparticles for self‐powered sweat electrolyte analysis. (f) Schematic diagram of self‐powered sweat analysis and its application in health monitoring. (g) Voltage of the electric nanogenerator as a function of sodium chloride (NaCl) concentration. (h) Voltage and conductivity of the electric nanogenerator as a function of NaCl concentration. Reproduced with permission [57]. Copyright 2023, Wiley‐VCH GmbH.

First, wastewater contains a variety of organic and inorganic contaminants from domestic, industrial, and commercial activities, resulting in different chemical properties [59]. In this context, wastewater can be utilized for hydrovoltaic power generation due to its specific chemical properties with various ions. Additionally, wastewater can also be treated during electricity generation, addressing environmental needs on both fronts. Jang et al. proposed a multifunctional membrane for simultaneous energy generation and water purification using wastewater [58]. The wastewater is purified as it passes through the electricity generation and purification membrane (EPM) in the perpendicular direction, while electricity is simultaneously generated in the horizontal direction by the movement of ions. The EPM can achieve high energy generation performance with a maximum power output of 16.44 µW, while also exhibiting water purification characteristics with >90% rejection of sub‐10 nm pollutants. Figure 7a shows the operation principle of the EPM system, demonstrating that only half of the EPM is exposed to wastewater, inducing asymmetric wetting for streaming‐based hydrovoltaic power generation. As polluted water passes vertically through the porous membrane of the EPM system, it undergoes purification. To verify the implementation of the EPM system, energy generation measurements were taken alongside water purification performance tests using industrial wastewater containing Trypan blue and PFOA. First, as polluted water passes through the EPM system, it maintains asymmetric wetting, inducing continuous voltage and current generation, with values of approximately 0.5 V and 75 µA, respectively (Figure 7b). Moreover, the EPM effectively filtered >80% of pollutants from industrial wastewater and demonstrated a high filtration efficiency of >93% for Trypan blue specifically (Figure 7c). The ability of the EPM system to simultaneously perform water purification and energy generation highlights its potential to address environmental issues such as water scarcity and eco‐friendly power generation. By utilizing wastewater as a specific resource, this approach provides a broader solution for various environmental challenges.

Second, seawater can also be a powerful resource for broadening the application of hydrovoltaic power generation. Seawater has high salinity and contains various ions [60] which can influence the electrical performance of large‐scale hydrovolatic power generation. In electrical double layer theory, the Debye length is influenced by ionic concentration, a key factor in hydrovoltaic power generation. As the ionic concentration increases, the reduction in the thickness of the electrical double layer enhances charge mobility, significantly increasing the generated current. Consequently, hydrovoltaic devices that utilize water sources with high ionic concentrations, such as seawater, demonstrate superior power characteristics. Thus, hydrovoltaic power generation using seawater holds considerable potential for high‐performance energy production. Li et al. proposed a dual‐mode power generation system that can harvest energy from dynamically changing aqueous solutions like seawater [16b]. Figure 7d shows the working mechanism of a flexible dual‐mode electricity nanogenerator (DM‐ENG). When the bottom electrode of the generator is placed in an aqueous solution, the solution climbs along the porous film under capillary force and evaporates at the interface between the hydrophilic and hydrophobic regions. The streaming potential can be enhanced through the ion concentration variation. Increasing ion concentration will reduce the streaming potential and increase the battery potential, especially in high‐salinity solutions (Figure 7e). In this context, saline solutions like seawater are useful for broadening the application to all dynamic salinity levels of most water environments.

Finally, biofluids such as blood, urine, and sweat contain specific biomarkers and numerous electrolytes, which can be utilized to indicate health status through specialized electricity generation [25, 61]. In this context, Huangfu et al. developed hydrovoltaic nanogenerators for self‐powered sweat electrolyte analysis [57]. The new concept of self‐powered sweat analysis through ion‐dependent hydrovoltaic power generation was proposed via sweat evaporation. This system can be used simultaneously as a continuous power supply and a sweat sensor for health monitoring (Figure 7f,g). Figure 7h shows that *V*_OC_ and *J*_SC_ exhibit a decreasing trend as the NaCl concentration increases from 10^−5^ to 10^−1^ mol L^−1^. The NaCl in the solution forms a charge shielding effect, reducing the Debye length, which leads to fewer anions accumulating on the top side of the device, resulting in a lower *V*_OC_. Therefore, the electrical performance of hydrovoltaic power generation changes proportionally to electrolyte levels in biofluids like sweat, resulting in the novel concept of self‐powered electrolyte sensors [62].

## 5 Applications

Hydrovoltaic power generation can be utilized in various applications due to its simple operation with water from sources such as sea, rivers, biofluids, and air [63]. With these diverse resources, hydrovoltaic power generation can serve as power sources, sensors, components of purification hybrid systems, and electrical stimulation devices for health therapy using biofluids. For each application and purpose, the power generation method and materials should be developed with careful consideration. To use hydrovoltaic power generation for power supply, its power efficiency needs enhancement [64]. For sensors and water purification, the focus should be on additional functions such as the linearity of sensing ability and purification capability. Additionally, for biomedical applications like electrical stimulation, biocompatible materials should be used [65]. With the flexibility of materials and simple processing mechanisms, hydrovoltaic power generation will be a powerful tool for various applications.

**FIGURE 8:**
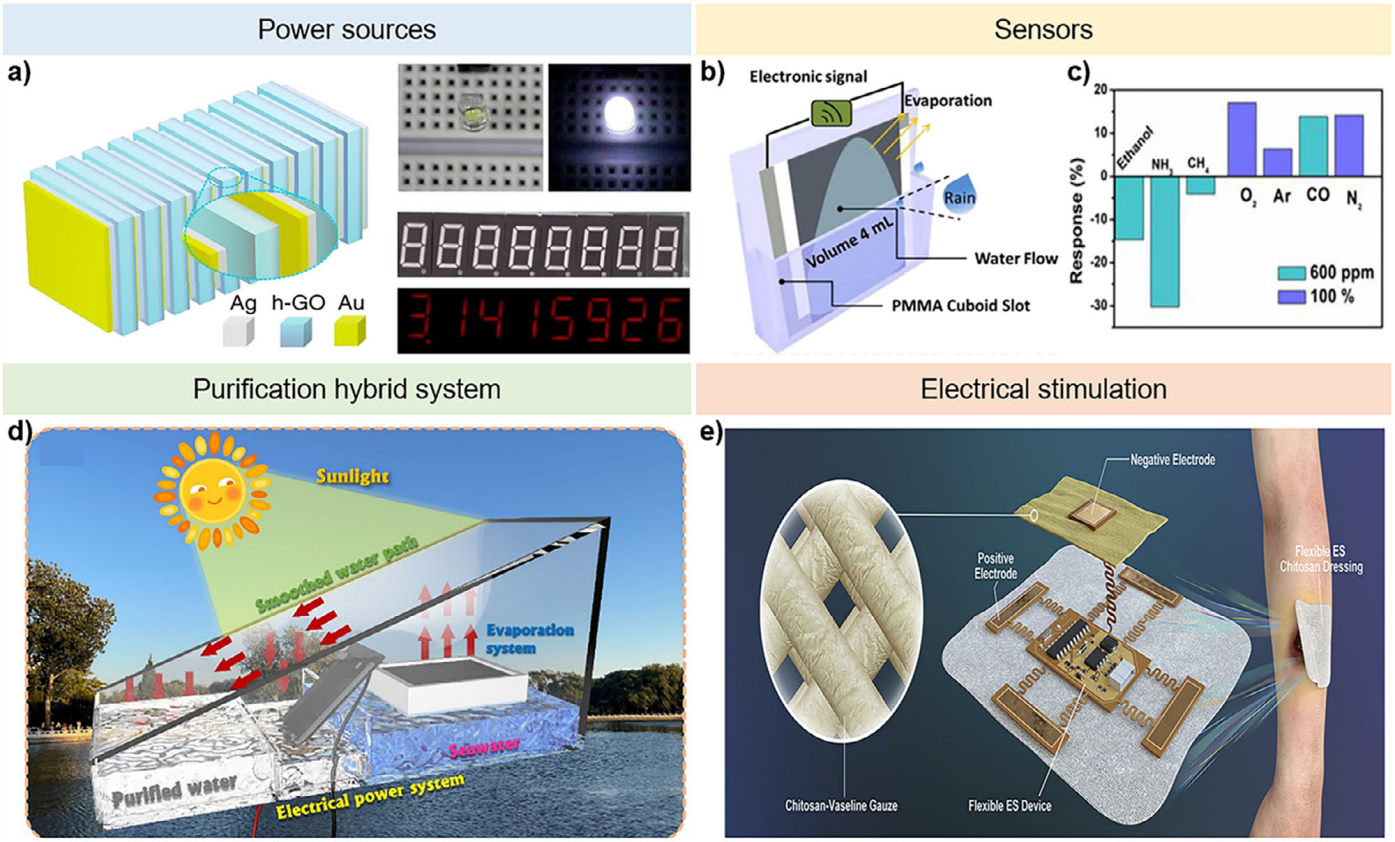
New perspectives on hydro(voltaic power generation. a) A high‐performance hygroelectric generator with bulk GO of a heterogeneous structure capable of driving commercial electronic devices. Reproduced with permission [56]. Copyright 2018, Springer Nature. (b,c) A novel self‐powered, flexibly‐arranged gas monitoring system using evaporating rainwater. (b) Schematic illustration of the self‐powered gas monitoring working model. (c) Response behavior of the gas sensor to different gases. Reproduced with permission [26]. Copyright 2019, Elsevier. (d) A solar‐driven seawater desalination and electricity generation integrated system with carbon‐black composites [27a]. Copyright 2023, Elsevier. (e) Schematic illustration of an electrical simulation device as a possible application. Reproduced with permission [27b]. Copyright 2021, Elsevier.

Hydrovoltaic power generation is primarily used as a power source, focusing on developing large‐scale power output [66]. For instance, Huang et al. proposed a high‐performance hygroelectric generator unit with an output voltage approaching 1.5 V, which can be easily scaled up by increasing the number of generator units to provide sufficient electricity for practical applications [56]. The device employs an effective synergy strategy involving the heterogeneous reconfiguration of oxygen‐containing functional groups on hygroscopic graphene oxide (GO) and mediation of electrode/material interfaces with well‐designed Schottky junctions. Figure 8a illustrates an assembled hygroelectric generator (HEG) package, incorporating Schottky and Ohmic contacts at the two ends of the heterogeneous graphene oxide (h‐GO), powering various commercial electronics through stored charge in a 1 µF capacitor. 15 HEG power units, using a layer‐by‐layer stacking strategy, generate a remarkable 18 V output and integrate with a commercial capacitor to demonstrate practical applications, successfully operating commercial electronics with the HEG package. Based on these results, they propose that the designed HEG has the potential to serve as a portable power source for self‐powered electronics.

Researchers are also exploring the electrical signals in hydrovoltaic power generation, leading to applications as sensors. In 2019, Zhong et al. proposed a novel self‐powered, flexibly arranged gas monitoring system based on a water‐evaporation/gas‐sensing coupling effect (Figure 8b) [26]. Rainwater evaporation along the device induces electricity, and the electrical output depends on the environmental atmosphere, making it suitable for gas sensing. The device can detect various gases such as oxygen, ethanol, ammonia, methane, carbon monoxide, argon, and nitrogen (Figure 8c). With this development, hydrovoltaic power generation has the potential to be used not only for self‐powering but also for broader applications as sensors.

As mentioned earlier, hydrovoltaic power generation can serve as a component of a purification system. Water, the primary resource for power generation, is closely related to the urgent challenge of freshwater shortages [67]. Therefore, there is a need to purify polluted water, and it would be beneficial if both energy and freshwater shortages could be addressed simultaneously [68]. In this context, Li et al. developed a novel type of solar‐driven interfacial evaporation and electricity generation integrated system for continuous seawater purification and hydrovoltaic power generation (Figure 8d) [27a]. They designed and synthesized a modified carbon black (MCB)‐decorated magnetic phase‐change composite (MCB‐MPCC) as a conductive photothermal material to induce water evaporation and electricity generation integrating system. The as‐synthesized MCB‐MPCC exhibits a high latent heat capacity of 145 J g^−1^, and the MCB‐MPCC‐based evaporator achieved a total water production of 2.63 kg m^−2^. Additionally, an electricity generator based on MCB‐MPCC was constructed with the developed evaporator, resulting in a stable output voltage of 0.43 V under sunlight illumination. This novel system suggests that hydrovoltaic power generation has a synergistic effect when combined with the purification process, which can be beneficial for sustainable seawater desalination and clean electric power generation.

Biofluid have the potential to serve as resources for hydrovoltaic power generation and can be utilized in biomedical applications such as wearable sensors [26] and electrical stimulation (Figure 8e). Specifically, electrical stimulation holds significant promise due to the simplicity of the device and the non‐toxic material used in hydrovoltaic power generation. With the advancement of wearable sensors utilizing biofluids, microcurrent electrical therapy emerges as a feasible application, like other energy‐harvesting nanogenerators [69]. For such applications, biocompatible materials are essential for wearable and implantable electronics used in electrical stimulation, and the power output should be tailored to meet specific treatment requirements, as different therapies may necessitate varying power densities [27, 70].

## 6 Summary and Perspective

Hydrovoltaic power generation represents an advanced technique for utilizing surface science. We have expanded upon this by providing mechanisms on various material types and case studies. This approach aims to offer readers deeper insights into the selection of materials for substrates and their potential future applications. Both hydrophilic and hydrophobic substrates can generate electrical power through flow or evaporation mechanisms. The novelty of hydrovoltaic power generation is demonstrated in Figure 6, which highlights the universal selection of materials that can be employed. This versatility has contributed to commercialization, but there are still many challenges that need to be overcome. The main issues lie in long‐term stability, high performance, efficiency, and water source management.

First, in terms of long‐term stability, there are problems such as corrosion caused by water, evaporation, and overall wetting from water flow, which make it difficult to maintain the system over time. Water energy harvesting inherently faces these issues due to the corrosive reactions with solids, the ease of water evaporation, and the movement of water as the primary energy source. To address these challenges, attempts have been made to use hygroscopic materials that continuously absorb water, to prevent corrosion and the depletion of energy resources. For water energy harvesting to be practically viable, it is necessary to resolve these problems while taking the characteristics of water into account.

Second, high performance is the most critical challenge that must be overcome for water energy harvesting to be used as a renewable energy source in the future. Innovative research on both device platforms and materials is needed to maximize the potential gradient between water and solid and to increase charge transfer. This requires an in‐depth understanding of the fundamental mechanisms, leading to the design of appropriate structures and materials. To achieve a deeper analysis of the mechanism, further research on operando characterization work for hydrovoltaic power generation is needed. Through operando characterization work, the system can be studied and analyzed in real‐time under operational conditions while interacting with water. This approach will allow for a more in‐depth understanding of the mechanism and electrical performance improvements.

Third, to improve efficiency, it is essential to minimize both the device area and the amount of water used, along with achieving high performance. This can be achieved by improving the nanomaterial structure to maximize contact with water and optimizing the overall platform design for better efficiency. Additionally, to minimize the amount of water used as an energy source, it is necessary to suppress water evaporation as much as possible and improve the system to deliver high performance with a minimal amount of water.

Last, from the perspective of securing water sources, there needs to be a discussion on environmental sustainability, given that water is an increasingly depleted resource. Hydrovoltaic devices can be powered by diverse water sources, including wastewater and seawater, which are not suitable for human consumption, as shown in Figure 7. Additionally, the potential for hydrovoltaic power generation arises from the abundance of water resources. The total volume of water on Earth is estimated to be approximately 1.4 × 10^9^ km^3^, with the annual evapotranspiration estimated at around 60,000 km^3^. This amount of evapotranspiration could theoretically operate a TEPG system 255.2 × 10^18^ times. This makes hydrovoltaic power one of the most reliable forms of renewable energy, positioning it as a high‐priority option for powering small electronic devices. Moreover, its capacity for installation in any location with access to water enables energy harvesting from various water sources, such as water surfaces, natural bodies of water, and water treatment facilities. This broad applicability has the potential to generate a substantial economic impact. This versatility underscores the broad applicability and potential of hydrovoltaic power generation. Thus, the perspective of this work is focused on sustainable energy generation, which is a key factor in sustainable development. In conclusion, we hope that hydrovoltaic technology becomes a viable option for powering devices.

## Conflicts of Interest Statement

The authors declare no conflicts of interest. Ho Won Jang is a member of the *Exploration* editorial board, and he was not involved in the handling or peer review process of this manuscript.
